# Diffuse idiopathic skeletal hyperostosis (DISH) of the elbow: a controlled radiological study

**DOI:** 10.1186/s12891-015-0575-5

**Published:** 2015-05-16

**Authors:** Christine Beyeler, Sergio R Thomann, Niklaus J Gerber, Christine Kunze, Daniel Aeberli

**Affiliations:** Department of Rheumatology, Immunology and Allergology, University Hospital, 3010 Bern, Switzerland; Assessment and Evaluation Unit, Institute of Medical Education, University of Bern, Bern, Switzerland

**Keywords:** Diffuse idiopathic skeletal hyperostosis (DISH), Elbow, Grading, Pathogenesis, Sex, Mechanical factors

## Abstract

**Background:**

Extraspinal manifestations of diffuse idiopathic skeletal hyperostosis (DISH) have been described previously. We aimed to assess the prevalence of elbow hyperostotic spurs, to search for sites discriminating for elbow DISH and to analyze the effect of physical activities, handedness and sex.

**Methods:**

Out of 284 patients hospitalized for extraskeletal disorders, 85 patients (33 with and 52 without thoracospinal DISH) agreed to bilateral elbow X-rays in two projections. Clinical information was collected by a standardized questionnaire and X-rays were graded blindly.

**Results:**

A total of 400 hyperostotic spurs (210 unilateral, 95 bilateral) were present at 11 predefined sites. The most frequent sites affected were the olecranon (20.8 %), lateral epicondyle (17.8 %) and medial epicondyle (15.5 %). In carriers of thoracospinal DISH significantly more hyperostotic spurs were present at the lateral and medial epicondyle compared to non-DISH carriers (OR 4.01 [95 % CI 1.35–12.34] and 2.88 [1.03–8.24], respectively). The olecranon, lateral and medial epicondyle contributed significantly to the classification of elbow DISH (OR 22.2 [4.1–144.7], 9.6 [1.9–61.2] and 10.1 [2.2–52.1], respectively). The prevalence of elbow hyperostotic spurs was higher in 45 patients with a history of heavy physical activities (24.4 % versus 18.0 %, OR 1.48 [1.17–1.86]), at the right elbow (24.2 % versus 18.6 %, OR 1.39 [1.11–1.75]) and in 62 males (22.8 % versus 17.6 %, OR 1.38 [1.06–1.81]).

**Conclusions:**

Hyperostotic spurs at the olecranon, lateral and medial epicondyle had the highest prevalence and disclosed the most pronounced discrimination for elbow DISH. Mechanical factors such as physical activities and handedness, and sex influenced the formation of these spurs.

**Electronic supplementary material:**

The online version of this article (doi:10.1186/s12891-015-0575-5) contains supplementary material, which is available to authorized users.

## Background

Diffuse idiopathic skeletal hyperostosis (DISH) is characterized by ossifications of entheses, where ligaments, tendons, joint capsules and annulus fibrosus fibres insert into bone. It involves the anterolateral aspect of the spine, but also several extraspinal sites, such as shoulder, elbow, hip, knee and heel [1–3]. The radiological findings of elbow hyperostosis have been described previously [3–11]. Elbow DISH is defined by the presence of both elbow and thoracospinal hyperostosis [8]. In carriers of thoracospinal hyperostosis the prevalence of elbow hyperostosis was shown to be increased about one and a half times compared to controls [8], pointing out that spinal and extraspinal manifestations might be features of an endocrine, metabolic or inflammatory disorder. Diseases associated with DISH are ankylosing spondylitis and related spondylarthropathies, acromegaly, hypertrophic osteoarthropathy, hypervitaminosis A, fluorosis, calcium pyrophosphate deposition disease, hyper- and hypoparathyroidism and ochronosis [2]. In addition, DISH is found in healthy individuals [2].

In the present study a detailed analysis of elbow hyperostotic spurs at predefined sites of both elbows was performed with the following aims 1) to assess the prevalence of elbow hyperostotic spurs at different sites, 2) to search for sites discriminating best for the presence of elbow DISH and 3) to analyze the effect of physical activities, handedness and sex.

## Methods

Consecutive routine lateral chest radiographs performed on patients admitted to internal medicine and cardiovascular surgery were screened for changes in the thoracic spine. Patients admitted for disorders related to the locomotor system, cancer, rheumatic, orthopedic or neurologic diseases were excluded after reviewing the medical records by one physician. Clinical information was collected by a blinded interviewer using a standardized questionnaire. Professional and unprofessional physical activities were classified as physically “heavy” or “light” by consensus of the two interviewers involved.

The lateral chest radiographs were graded blindly by a rheumatologist according to the following classification [12, 13]: Grade 0: no ossification; Grade I: prevertebral and/or prediscal ossification at one or two vertebral bodies of the spine or one bridging ossification; Grade II: flowing continuous prediscal and/or prevertebral ossification along three or more vertebral bodies or two bridging ossifications; Grade III: three or more bridging prediscal or prevertebral ossifications. The intervertebral discs of the hyperostotic segments were not allowed to show any degenerative, inflammatory or dysplastic abnormalities [12, 13]. Grades 0 and I were classified as “thoracospinal DISH absent”; grades II and III as “thoracospinal DISH present”.

The bilateral elbow X-rays with anterior-posterior and lateral views were graded blindly and independently by a rheumatologist and a radiologist according to the following classification [8]: Grade 0: none or only one ossification attached to bone of less than 2 mm; Grade I: two or more ossifications of less than 2 mm or one ossification of 2–3 mm; Grade II: two or more ossifications of more than 2 mm or one ossification of more than 3 mm; Grade III: two or more ossifications of more than 3 mm. Grades 0 and I were classified as “elbow hyperostosis absent”, grades II and III as “elbow hyperostosis present”. “Elbow DISH” was defined by the presence of both thoracospinal and elbow hyperostosis [8]. In addition, the presence of other skeletal changes such as chondrocalcinosis, inflammatory or degenerative features, and amorphous soft tissue calcifications were noted. Analyses of intra- and interobserver reliability of the spinal and elbow grading were performed as published previously [8, 12].

Statistical analyses included the calculation of odds ratios (OR), 95 % CIs and logistic procedures with step down regression analyses with four independent variables. The statistical programs used comprised Epi Info5 (USD, Georgia, USA) and Statistical Analysis System (SAS Institute Inc., Cary, NC, USA) under license of the University of Bern.

The ethics committee of the University of Bern, Switzerland approved the study and consent was obtained from all patients.

## Results

A total of 284 age- and sex-matched patients with and without thoracospinal DISH were included into the study. Eighty-five patients agreed to a complete radiological examination of the thoracic spine and both elbows. Their agreement to elbow X-rays was independent of thoracospinal DISH [8]. Their mean age was 67 ± SD 9.2 years, 62 (72.9 %) were male and 45 (52.9 %) gave a history of heavy physical activities.

**Fig. 1:**
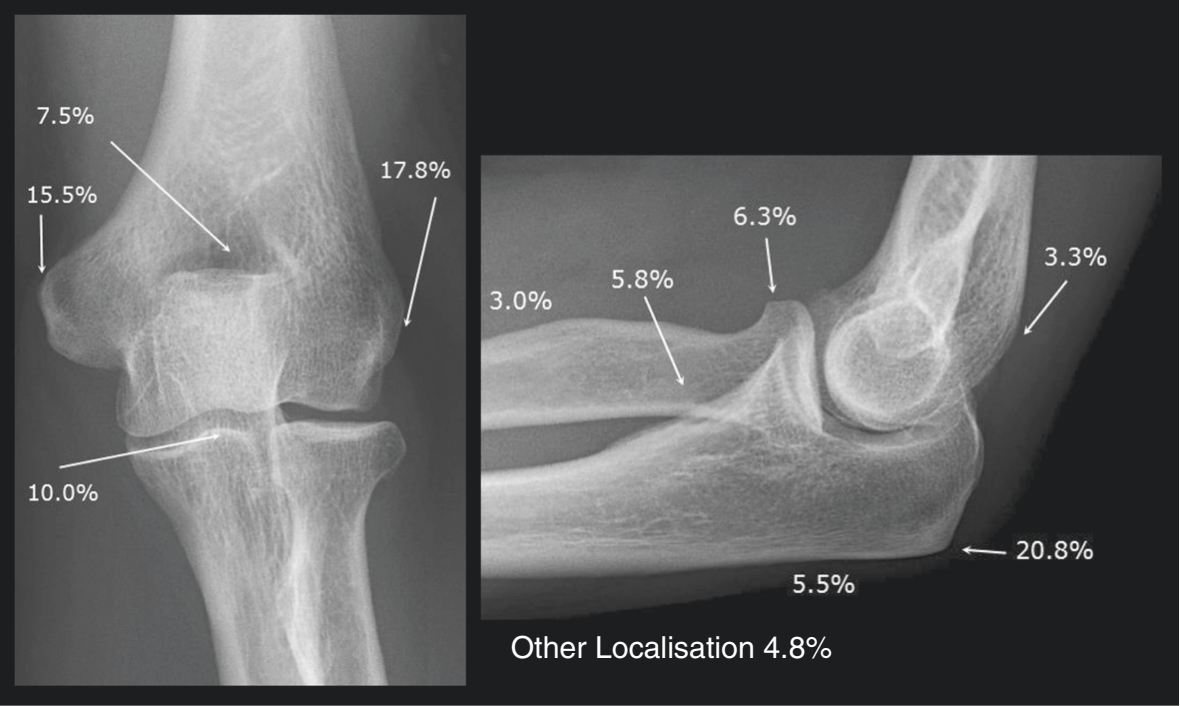
Frequency distribution of the 400 hyperostotic spurs detected at all localizations of both elbows, irrespective of thoracospinal and elbow grading, in 85 patients hospitalized for disorders not related to the locomotor system

Radiological classification revealed thoracospinal DISH to be present in 33 (38.8 %), absent in 52 (61.2 %); elbow hyperostosis present in 57 (67.1 %), absent in 28 (32.9 %); elbow DISH (defined by the features of both thoracospinal and elbow hyperostosis) present in 27 (31.8 %) and absent in 22 (25.9 %) patients. Detailed investigation of 11 predefined localizations of both elbows showed 210 unilateral and 95 bilateral hyperostotic spurs resulting in a total of 400 out of 1870 (21.3 %) possible hyperostotic spurs. Frequency distribution of these documented hyperostotic spurs is illustrated in Fig. 1. Prevalence at specified sites was as follows: olecranon 48.8 % (83 out of 170 possible spurs), lateral epicondyle 41.8 %, medial epicondyle 36.5 %, coronoid process 23.5 %, coronoid fossa 17.6 %, radial head 14.7 %, radial tuberosity 13.5 %, olecranon other localization 12.9 %, olecranon fossa 7.6 % and radius other localization 7.1 %, respectively.

In patients with *thoracospinal DISH* significantly more hyperostotic spurs were found at the lateral epicondyle (78.8 % versus 48.1 %, OR 4.01 [1.35–12.34]) and medial epicondyle (72.7 % versus 48.1 %, OR 2.88 [1.03–8.24]) compared to patients without thoracospinal hyperostosis. However, the total number of hyperostotic spurs at all sites was similar between the two groups (35.5 % versus 30.8 %, OR 1.24 [0.93–1.66]). In addition, no significant differences were found at all other predefined localizations (Table 1).

**Table 1:** Hyperostotic spurs at 11 specified localizations of both elbows in patients with or without thoracospinal DISH, with or without elbow hyperostosis, and with or without elbow DISH (defined by the features of both thoracospinal and elbow hyperostosis), respectively

	Thoracospinal hyperostosis			Elbow hyperostosis			Elbow DISH (Elbow and Thoracospinal Hyperostosis)		
	Present (n = 33)	Absent (n = 52)	OR (95 % CI)	Present (n = 57)	Absent (n = 28)	OR (95 % CI)	Present (n = 27)	Absent (n = 22)	OR (95 % CI)
Olecranon	66.7 %	57.7 %	1.47 (0.54–4.03)	84.2 %	14.3 %	**32.00** (7.73–148.86)	77.8 %	13.6 %	**22.17** (4.13–144.69)
Lateral epicondyle	78.8 %	48.1 %	**4.01** (1.35–12.34)	68.4 %	21.1 %	**2.89** (1.03–8.20)	88.9 %	45.5 %	**9.60** (1.91–61.23)
Medial epicondyle	72.7 %	48.1 %	**2.88** (1.03–8.24)	70.2 %	15.8 %	**4.97** (1.70–14.91)	85.2 %	36.4 %	**10.06** (2.18–52.12)
Coronoid process	39.4 %	38.5 %	1.04 (0.39–2.79)	49.1 %	8.8 %	**4.44** (1.37–16.79)	48.1 %	22.7 %	3.16 (0.79–13.91)
Coronoid fossa	21.2 %	32.7 %	0.55 (0.18–1.69)	35.1 %	7.0 %	3.24 (0.91–14.48)	25.9 %	18.2 %	1.58 (0.33–8.53)
Radial head	24.2 %	23.1 %	1.07 (0.34–3.32)	29.8 %	5.3 %	3.54 (0.88–20.49)	29.6 %	13.6 %	2.67 (0.52–17.65)
Radial tuberosity	30.3 %	19.2 %	1.83 (0.59–5.66)	28.1 %	7.0 %	2.34 (0.64–10.65)	29.6 %	9.1 %	4.21 (0.69–44.44)
Olecranon - other localization	12.1 %	26.9 %	0.37 (0.08–1.38)	24.6 %	7.0 %	1.95 (0.53–9.01)	14.8 %	18.2 %	0.78 (0.13–4.85)
Other localization	21.2 %	19.2 %	1.13 (0.34–3.77)	24.6 %	5.3 %	2.71 (0.66–15.98)	25.9 %	13.6 %	2.22 (0.42–15.00)
Olecranon fossa	12.1 %	15.4 %	0.76 (0.15–3.16)	35.1 %	7.0 %	3.24 (0.91–14.48)	14.8 %	13.6 %	1.10 (0.16–8.45)
Radius - other localization	12.1 %	9.6 %	1.30 (0.24–6.56)	12.3 %	3.5 %	1.82 (0.31–19.06)	11.1 %	4.5 %	2.63 (0.19–144.25)

Significant values with *p* < 0.05 are reported in bold

In patients with *elbow hyperostosis* significantly more hyperostotic spurs were discovered at the olecranon (84.2 % versus 14.3 %, OR 32.0 [7.7–148.9]), medial epicondyle (70.2 % versus 15.8 %, OR 4.97 [1.70–14.91]), coronoid process (49.1 % versus 8.8 %, OR 4.44 [1.37–16.97]) and lateral epicondyle (68.4 % versus 21.1 %, OR 2.89 [1.03–8.20]), compared to patients without elbow hyperostosis. No significant differences were found at all other predefined localizations (Table 1).

In patients with *elbow DISH* significantly more hyperostotic spurs were detected at the olecranon (77.8 % versus 13.6 %, OR 22.17 [4.13–144.69]), medial epicondyle (85.2 % versus 36.4 %, OR 10.06 [2.18–52.12]) and lateral epicondyle (88.9 % versus 45.5 %, OR 9.60 [1.91–61.23]), compared to patients without elbow DISH. No significant differences were found at all other predefined localizations (Table 1).

The total number of elbow hyperostotic spurs was significantly higher in patients with a history of heavy physical activities (24.4 % versus 18.0 %, OR 1.48 [1.17–1.86]), on the right side (24.2 % versus 18.6 %, OR 1.39 [1.11–1.75]) and in males (22.8 % versus 17.6 %, OR 1.38 [1.06–1.81]).

Multiple logistic regression analyses of the presence of elbow hyperostotic spurs with stepdown regression for four independent variables (thoracospinal hyperostosis, age, physical activities, sex) confirmed the sex difference with significantly more hyperostotic spurs in males at the right and left olecranon (OR 2.96 [1.07–8.22] and 7.03 [2.14–23.15], respectively). The complete results are presented in the Additional file 1.

## Discussion

Herein we analyzed the prevalence of hyperostotic spurs at 11 predefined sites of both elbows in patients with or without thoracospinal hyperostosis hospitalized for disorders not related to the locomotor system. To our knowledge this is the most detailed study of the extraspinal involvement of DISH at the elbow.

In our study, the prevalence of elbow hyperostotic spurs in carriers of thoracospinal DISH was similar to findings in smaller series of 5 to 27 individuals with percentages ranging from 42 % to 81 % at the olecranon and 29 % to 81 % at unspecified sites of the elbow [3–7, 11]. However, the prevalence of elbow hyperostotic spurs in patients without thoracospinal DISH was higher than the percentage of 10 % described in another controlled study [4].

A considerably high proportion of patients without thoracospinal manifestations showed hyperostotic spurs at the olecranon, lateral and medial epicondyle. The reason for this is unknown. Various diseases where ligamentous or capsular ossifications can occur [2] have deliberately been excluded in our study. Possible explanations are that extraspinal sites might be involved before the spine [2], or that chronic mechanic overloading and tear might lead to osteoanabolic reaction at insertion of the tendon to the bone. Thus, our findings support the hypothesis that mechanical factors play an important role in the development of hyperostotic spurs. First, hyperostotic spurs were found almost one and a half times more often on the right side where the majority of patients was expected to be right-handed. Second, hyperostotic spurs were almost one and a half times more prevalent in patients with a history of heavy professional or unprofessional physical activities. Third, hyperostotic spurs were present particularly at the interface between strong muscles and bone, such as triceps (olecranon), extensor carpi radialis brevis and longus (lateral epicondyle), and pronator teres and flexor carpi radialis muscle (medial epicondyle), respectively. Similar influence has been described at the shoulder, where the highest prevalence of hyperostotic spurs was found at the insertion of the biceps long head, triceps brachii (glenoid), supraspinatus, infraspinatus, teres minor (greater tuberculum), and deltoideus muscle (acromion), respectively [14]. This is also in agreement with findings at the patella, the tibial tuberositas and the calcaneus [4, 5, 7]. In our study we found one and a half times more hyperostotic spurs in males than in females. This was confirmed for the olecranon by a multiple logistic regression analysis taking into account the potential confounding factors age, physical activities and the presence of thoracospinal hyperostosis. These findings underline the hypothesis, that hormonal factors influence the growth of spurs. Estrogens might have a protective effect. DISH is more prevalent in men, and the incidence increases with age [15]. The protective effect of estrogens on the development of DISH seems to be of metabolic nature, since differences of volumetric bone density and osteoblast activity have not been seen in quantitative bone scans or functional Dickkopf-1 serum levels [16–18]. This hormonal influence is in line with associations of DISH with obesity, diabetes, hyperlipidemia, gout, hypertension and coronary artery disease as described previously [2, 9, 19, 20].

The investigation of the clinical relevance of elbow hyperostosis was not the focus of this study. As has been shown previously carriers of elbow hyperostosis developed elbow pain only slightly more frequently compared to controls [8]. However, shoulder hyperostosis irrespective of or in combination with thoracospinal hyperostosis DISH predisposed to shoulder pain two to four times [21]. In individuals with heavy professional or unprofessional physical activities ergonomic adaptations, aptitude counselling and occupational therapy measures might be of particular relevance if extraspinal involvement of DISH is present. Further studies in occupational medicine could address the long-term ability to perform physical activities at work or for leisure in the presence or absence of DISH at the elbow and other extraspinal sites if prophylactic measures are taken.

### Limitations of the study

Since X-rays of the sacroiliacal joints were not systematically performed in all patients osteoanabolic changes related to spondylarthropathies cannot be ruled out. However, it is unlikely, that a patient with ankylosing spondylitis or any other axial spondylarthitis was missed by the careful screening of the individual medical records.

Multiple statistical testing was performed and intracluster correlations within patients were not taken into account. However, the consistency of the findings at the olecranon, lateral and medial epicondyle and the confirmation by multiple logistic regression analyses reduced the risk of misinterpretation of false positive results. The analyses of the overall effects of physical activities, handedness and sex revealed positive findings and were a main focus of the study. In contrast, the subanalyses of these factors at the different sites were mostly negative as could be expected in view of the limited power of the study. Due to the lack of immediate clinical relevance the value of a study with a higher number of patients is questionable.

## Conclusion

Our results underline the multifactorial nature of DISH with mechanical and hormonal factors playing a role in the ossifications of entheses.

## Additional file

Additional file 1:
**Logistic regression analyses of the presence of elbow hyperostotic spurs at 11 specified sites with stepdown regression for four independent variables (thoracospinal hyperostosis, age, physical activities, sex).** ^*^ *p*-value (Wald Chi-Square); ^**^95 % CI; ^***^missing values: no SAS output, the maximum likelihood estimate may not exist. Significant ORs with *p* < 0.05 are reported in bold.

## Abbreviations

CI: Confidence interval
DISH: Diffuse idiopathic skeletal hyperostosis
OR: Odds ratio
SD: Standard deviation

## References

[1] Forestier J, Rotes-Querol J (1950). Senile ankylosing hyperostosis of the spine. Ann Rheum Dis.

[2] Resnick D, Shapiro RF, Wiesner KB, Niwayama G, Utsinger PD, Shaul SR (1978). Diffuse idiopathic skeletal hyperostosis (DISH) [ankylosing hyperostosis of Forestier and Rotes-Querol]. Semin Arthritis Rheum.

[3] Arlet J, Mazieres B. [Hyperostotic disease]. La Revue de medecine interne/fondee par la Societe nationale francaise de medecine interne. 1985;6(5):553–64. 10.1016/s0248-8663(85)80037-0 3914022

[4] Resnick D, Shaul SR, Robins JM (1975). Diffuse idiopathic skeletal hyperostosis (DISH): Forestier’s disease with extraspinal manifestations. Radiology.

[5] Littlejohn GO, Urowitz MB (1982). Peripheral enthesopathy in diffuse idiopathic skeletal hyperostosis (DISH): a radiologic study. J Rheumatol.

[6] Robotti GC, Schneekloth G (1982). [Extravertebral manifestations of ankylosing hyperstosis (Forestier’s disease)]. Radiologe.

[7] Doyle TC, Littlejohn G (1986). The radiological features of diffuse idiopathic skeletal hyperostosis (D.I.S.H.). Australas Radiol.

[8] Beyeler C, Schlapbach P, Gerber NJ, Fahrer H, Hasler F, van der Linden SM (1992). Diffuse idiopathic skeletal hyperostosis (DISH) of the elbow: a cause of elbow pain? A controlled study. Br J Rheumatol.

[9] Mata S, Fortin PR, Fitzcharles MA, Starr MR, Joseph L, Watts CS (1997). A controlled study of diffuse idiopathic skeletal hyperostosis. Clinical features and functional status. Medicine.

[10] Mata S, Chhem RK, Fortin PR, Joseph L, Esdaile JM (1998). Comprehensive radiographic evaluation of diffuse idiopathic skeletal hyperostosis: development and interrater reliability of a scoring system. Semin Arthritis Rheum.

[11] Mazieres B, Rovensky J (2000). Non-inflammatory enthesopathies of the spine: a diagnostic approach. Bailliere’s Best Pract Res Clin Rheumatol.

[12] Schlapbach P, Beyeler C, Gerber NJ, van der Linden S, Burgi U, Fuchs WA (1989). Diffuse idiopathic skeletal hyperostosis (DISH) of the spine: a cause of back pain? A controlled study. Br J Rheumatol.

[13] Resnick D, Niwayama G (1976). Radiographic and pathologic features of spinal involvement in diffuse idiopathic skeletal hyperostosis (DISH). Radiology.

[14] Beyeler C, Lehmann T, Schlapbach P, Gerber NJ, Fuchs WA (1995). Diffuse idiopathic skeletal hyperostosis (DISH) of the shoulder. A controlled radiological study. Rheumatol Int.

[15] Nascimento FA, Gatto LA, Lages RO, Neto HM, Demartini Z, Koppe GL (2014). Diffuse idiopathic skeletal hyperostosis: A review. Surg Neurol Int.

[16] Eser P, Bonel H, Seitz M, Villiger PM, Aeberli D (2010). Patients with diffuse idiopathic skeletal hyperostosis do not have increased peripheral bone mineral density and geometry. Rheumatology (Oxford).

[17] Aeberli D, Schett G, Eser P, Seitz M, Villiger PM (2011). Serum Dkk-1 levels of DISH patients are not different from healthy controls. Joint Bone Spine.

[18] Senolt L, Hulejova H, Krystufkova O, Forejtova S, Andres Cerezo L, Gatterova J (2012). Low circulating Dickkopf-1 and its link with severity of spinal involvement in diffuse idiopathic skeletal hyperostosis. Ann Rheum Dis.

[19] Zincarelli C, Iervolino S, Di Minno MN, Miniero E, Rengo C, Di Gioia L (2012). Diffuse idiopathic skeletal hyperostosis prevalence in subjects with severe atherosclerotic cardiovascular diseases. Arthritis Care Res.

[20] Kiss C, Szilagyi M, Paksy A, Poor G (2002). Risk factors for diffuse idiopathic skeletal hyperostosis: a case–control study. Rheumatology.

[21] Beyeler C, Schlapbach P, Gerber NJ, Sturzenegger J, Fahrer H, van der Linden S (1990). Diffuse idiopathic skeletal hyperostosis (DISH) of the shoulder: a cause of shoulder pain?. Br J Rheumatol.

