# Supplementary material for: Diffuse idiopathic skeletal hyperostosis (DISH) of the elbow: a controlled radiological study
Source: BMC Musculoskelet Disord. 2015 May 16;16:119. doi: 10.1186/s12891-015-0575-5 (PMC4493825; doi:10.1186/s12891-015-0575-5)
Supplement: Additional file 1: — Logistic regression analyses of the presence of elbow hyperostotic spurs at 11 specified sites with stepdown regression for four independent variables (thoracospinal hyperostosis, age, physical activities, sex). * p-value (Wald Chi-Square); **95 % CI; ***missing values: no SAS output, the maximum likelihood estimate may not exist. Significant ORs with p < 0.05 are reported in bold. [file 12891_2015_575_MOESM1_ESM.docx]

**Additional file 1**

| Localization of the  Hyperostotic Spurs  (n = number present) | Thoracospinal Hyperostosis (present versus absent) | Physical Activities  (yes versus no) | Age  (older versus jounger) | Sex  (male versus female) |
| --- | --- | --- | --- | --- |
| Olecranon [right] (n=42) | 0.542 | 0.568 | 0.138 | 0.037 ^*^  **OR = 2.96**  1.07-8.22 ^**^ |
| Olecranon [left] (n=41) | 0.565 | 0.102 | 0.944 | 0.001  **OR = 7.03**  2.14-23.15 |
| Lateral epicondyle [right] (n=39) | 0.003  **OR = 4.59**  1.66-12.68 | 0.016  **OR = 3.41**  1.26-9.27 | 0.617 | 0.889 |
| Lateral epicondyle [left] (n=32) | 0.038  **OR = 2.71**  1.06-6.95 | 0.292 | 0.033  **OR = 1.06**  1.01-1.12 | 0.917 |
| Medial epicondyle [right] (n=36) | 0.175 | 0.121 | 0.977 | 0.435 |
| Medial epicondyle [left] (n=26) | 0.001  **OR = 5.08**  1.88-13.68 | 0.923 | 0.396 | 0.570 |
| Coronoid process [right] (n=20) | 0.939 | 0.382 | 0.011  **OR = 1.09**  1.02-1.16 | 0.628 |
| Coronoid process [left] (n=20) | 0.772 | 0.298 | 0.434 | 0.065 |
| Coronoid fossa [right] (n=22) | 0.675 | 0.246 | 0.946 | 0.333 |
| Coronoid fossa [left] (n=8) | 0.302 | 0.545 | 0.630 | 0.349 |
| Radial head [right] (n=17) | 0.416 | 0.110 | 0.496 | 0.224 |
| Radial head [left] (n=8) | 0.398 | 0.786 | 0.396 | 0.501 |
| Radial tuberosity [right] (n=13) | 0.375 | 0.280 | 0.199 | 0.964 |
| Radial tuberosity [left] (n=10) | - | - | - | - ^***^ |
| Olecranon – other localization [right] (n=15) | 0.422 | 0.090 | 0.841 | 0.915 |
| Olecranon – other localization [left] (n=7) | - | - | - | - |
| Other localization [right] (n=8) | 0.869 | 0.474 | 0.681 | 0.349 |
| Other localization [left] (n=11) | - | - | - | - |
| Olecranon fossa [right] (n=6) | 0.447 | 0.202 | 0.136 | 0.745 |
| Olecranon fossa [left] (n=7) | - | - | - | - |
| Radius – other localization  [right] (n=8) | - | - | - | - |
| Radius – other localization  [left] (n=4) | - | - | - | - |
